# Interpretable Independent Recurrent Networks for Forecasting Stroke in Atrial Fibrillation

**DOI:** 10.1016/j.jacasi.2025.04.003

**Published:** 2025-06-10

**Authors:** Jung-Chi Hsu, Yi-Hsien Hsieh, Yen-Yun Yang, Shu-Lin Chuang, Che Lin, Lian-Yu Lin

**Affiliations:** a Department of Internal Medicine, National Taiwan University Hospital Jinshan Branch, New Taipei City, Taiwan; b Division of Cardiology, Department of Internal Medicine, National Taiwan University Hospital and College of Medicine, Taipei, Taiwan; c Graduate Institute of Communication Engineering, National Taiwan University, Taipei, Taiwan; d Department of Medical Research, National Taiwan University Hospital, Taipei, Taiwan; e Department of Electrical Engineering, National Taiwan University, Taipei, Taiwan; f Center for Advanced Computing and Imaging in Biomedicine, National Taiwan University, Taipei, Taiwan; g Smart Medicine and Health Informatics Program, National Taiwan University, Taipei, Taiwan; h Cardiovascular Center, National Taiwan University Hospital, Taipei, Taiwan; i Department of Internal Medicine, National Taiwan University Hospital, Yunlin Branch, Yunlin, Taiwan

**Keywords:** atrial fibrillation, explainable artificial intelligence, gated recurrent unit, recurrent neural network (RNN), stroke

## Abstract

**Background:**

Atrial fibrillation (AF) is a major risk factor for transient ischemic attack (TIA)/ischemic stroke (IS).

**Objectives:**

Given the dynamic nature of IS risk, this study aimed to predict IS risk in AF patients using a high-dimensional time-series model.

**Methods:**

We conducted a cohort study at the National Taiwan University Hospital from 2014 to 2019, including 7,710 AF patients, with external validation in 6,822 patients from the National Taiwan University Hospital Yunlin Branch. The Forecasting Strokes via Interpretable Independent Networks (ForeSIIN) model, based on gated recurrent units, was proposed. Kaplan-Meier analysis with log-rank test evaluated risk group differences.

**Results:**

The annual TIA/IS incidence rate ranged from 181.96 (95% CI: 164.42-200.93) to 15.81 (95% CI: 12.38-20.18) per 1,000 person-years, with an overall incidence of 42.40 (95% CI: 39.60-45.39). The ForeSIIN model achieved the best prediction with an area under the receiver-operating characteristics curve of 0.764 (95% CI: 0.722-0.810), compared with the CHA_2_DS_2_-VASc score (AUC: 0.650; 95% CI: 0.596-0.699) and other nonsequential models: extreme gradient boosting AUC: 0.722 (95% CI: 0.676-0.769), support vector machine AUC 0.691 (95% CI: 0.637-0.741), random forest AUC: 0.689 (95% CI: 0.637-0.742). External validation showed area under the receiver-operating characteristics curve of 0.646 (95% CI: 0.618-0.671) and area under the precision-recall curve of 0.222 (95% CI: 0.184-0.259). Feature impact analysis identified the top 5 factors: history of TIA/IS, estimated glomerular filtration rate, C-reactive protein, hematocrit, and plasma fasting glucose. Kaplan-Meier analysis showed significant risk differences between ForeSIIN groups (log-rank *P <* 0.001).

**Conclusions:**

The innovative ForeSIIN model demonstrated accurate stroke prediction in AF patients and enhanced the interpretation of dynamic risk factors over time.

Atrial fibrillation (AF) is the most common arrhythmia associated with increased comorbidities and mortality.^1^^,^^2^ Structural remodeling of the left atrium and hypercoagulability contribute to AF onset and persistence, leading to atrial cardiomyopathy. AF-related strokes, often caused by left atrial appendage thrombus formation, have worse prognoses compared with strokes from other causes.^3^ Early anticoagulant therapy can prevent AF-associated thromboembolism, necessitating stroke risk scores for anticoagulant evaluation.^4^ Various risk factors have been identified and integrated into stroke risk stratification methods.

Current risk prediction models, such as AFI, SPAF, Framingham Heart Study, and CHADS_2_ score, demonstrate fair discriminatory power (c-statistics: 0.56-0.60).^5^ The CHA_2_DS_2_-VASc score is recommended in anticoagulation guidelines,^6^, ^7^, ^8^ but its superiority over the CHADS_2_ score for thromboembolism is evident in real-world cohorts. ATRIA (Anticoagulation and Risk Factors in Atrial Fibrillation) risk score improves ischemic stroke (IS) prediction but with modest discrimination (c-statistics: 0.68-0.70).^9^^,^^10^ Recent studies suggest that incorporating biomarkers and echocardiographic parameters may enhance stroke risk classification in AF. Prediction models, including relevant echocardiographic variables in stroke scoring systems or as predictive factors, could provide a better representation of atrial cardiomyopathy in these patients.^11^^,^^12^

Stroke risk prediction in AF is dynamic, evolving over time because of aging and the accumulation of comorbidities, as highlighted by nationwide epidemiological studies.^13^ This progression complicates risk assessment, because traditional models like the CHA_2_DS_2_-VASc score, which rely solely on baseline characteristics, may underestimate the true stroke risk in AF patients whose clinical profiles change over time.

Machine learning (ML) has shown considerable promise in the field of AF. These advancements are largely driven by the application of sophisticated algorithms to diverse data modalities, such as electrocardiogram (ECG) and brain diffusion-weighted imaging.^14^^,^^15^ In a large cohort of newly diagnosed, non-anticoagulated AF patients, significant improvements in stroke risk prediction were observed through the use of ML analysis. This was accompanied by favorable net benefit values and strong internal and external calibration.^16^^,^^17^ However, existing ML models that incorporate comorbidities and health records often rely on cross-sectional data,^18^ which are prone to issues such as noise and missing values, limiting their effectiveness for dynamic risk prediction.

In contrast, recurrent neural networks (RNNs) have been increasingly used in AF research, particularly for predicting the onset of AF through ECG beat-to-beat interval time series or portable ECG recordings.^19^, ^20^, ^21^ Despite these advancements, their application for predicting stroke risk or other clinical outcomes in AF patients remains underexplored. This study seeks to fill that gap by employing RNNs to analyze the dynamic progression of stroke risk in AF patients over time. To this end, we developed the Forecasting Strokes via Interpretable Independent Networks (ForeSIIN), a novel deep-learning RNN model designed to capture the temporal evolution of stroke risk in AF patients using time-series data.

## Methods

### Study population

A longitudinal retrospective cohort study was conducted from January 1, 2014, to December 31, 2019, utilizing electronic health records (EHRs) from Taiwanese individuals in the Integrative Medical Data Center, National Taiwan University Hospital (NTUH-iMD). Since 2006, EHRs have been digitized and made available online. These records encompass a comprehensive range of patient data, including diagnoses, physician prescriptions, laboratory results, interventions, medications, and examinations. Previous research has demonstrated the validity of these medical data for providing real-world evidence.^22^ The NTUH-iMD includes data from both the main hospital in Taipei and independent local branches in central and southern Taiwan. Additionally, an independent cohort was collected from the NTUH Yunlin Branch for external validation. The research was approved by the Institutional Review Board of the National Taiwan University Hospital (approval number: 202403114RINC). The study follows the TRIPOD guidelines for reporting predictive model development and validation.

We enrolled AF patients during the follow-up. The International Classification of Diseases codes from the EHRs or conventional 12-lead ECGs were used to identify AF and its occurrence time. At least 1 electrophysiologist reviewed the electrocardiograms and confirmed the AF or atrial flutter diagnosis. The World Health Organization endorses the International Classification of Diseases-10th Revision code for AF and atrial flutter. Anticoagulant medicine was administered in accordance with the current AF treatment guideline. This study's endpoint was the occurrence of transient ischemic attack (TIA) or IS following the first AF event. A central committee determined the cause of death. We evaluated all accessible medical data until the last clinical visit or death, whichever came first. Every patient has at least 1 check record after their first AF episode. No patient was excluded from the input sequence or label construction.

We evaluated baseline characteristics, including age, sex, body mass index, history of hypertension, hyperlipidemia, gout, heart failure, coronary artery disease, chronic kidney disease, chronic obstructive pulmonary disease, peripheral arterial occlusive disease, and TIA/IS, using EHR data. The extracted laboratory data included liver function tests, including aspartate aminotransferase and alanine aminotransferase, and renal function tests, including blood urea nitrogen and creatinine, N-terminal pro–B-type natriuretic hormone level, high-sensitivity C-reactive protein (hsCRP), hematocrit, blood glucose level, and lipid profile. The estimated glomerular filtration rate (eGFR) was calculated using the Modification of Diet in Renal Disease equation, which adjusts for serum creatinine, age, sex, and race: eGFR = 175 × (serum Cr; mg/dL)^−1.154^× age^−0.203^ × 1.212 (if the patient is black) × 0.742 (if female).^23^

Echocardiographic tests were conducted, and 2-dimensional, M-mode measurements were taken with a 3.0 or 3.5 MHz transducer. Using the M-mode cursor, we collected left atrium size, left ventricular internal dimension in end-diastole and systole, and left ventricular ejection function values from the parasternal long-axis view. Left ventricular mass (LVM) was calculated using Devereux's formula: 0.8(1.04[([LVEDD + IVSd + PWd]^3^ − LVEDD^3^)]) + 0.6, where LVEDD, IVSd, and PWd represent LV end-diastolic diameter, interventricular septal thickness in diastole, and posterior wall thickness in diastole, respectively, and was derived assuming LV dimensions in centimeters.^24^ The LVM index was derived by dividing the LVM by body surface area. All echocardiographic data was compiled from the EHRs. All characteristics, the underlying disease, laboratory investigations, and echocardiogram parameters were entered into our sequence as features.

### Data preprocessing

**Figure 1:**
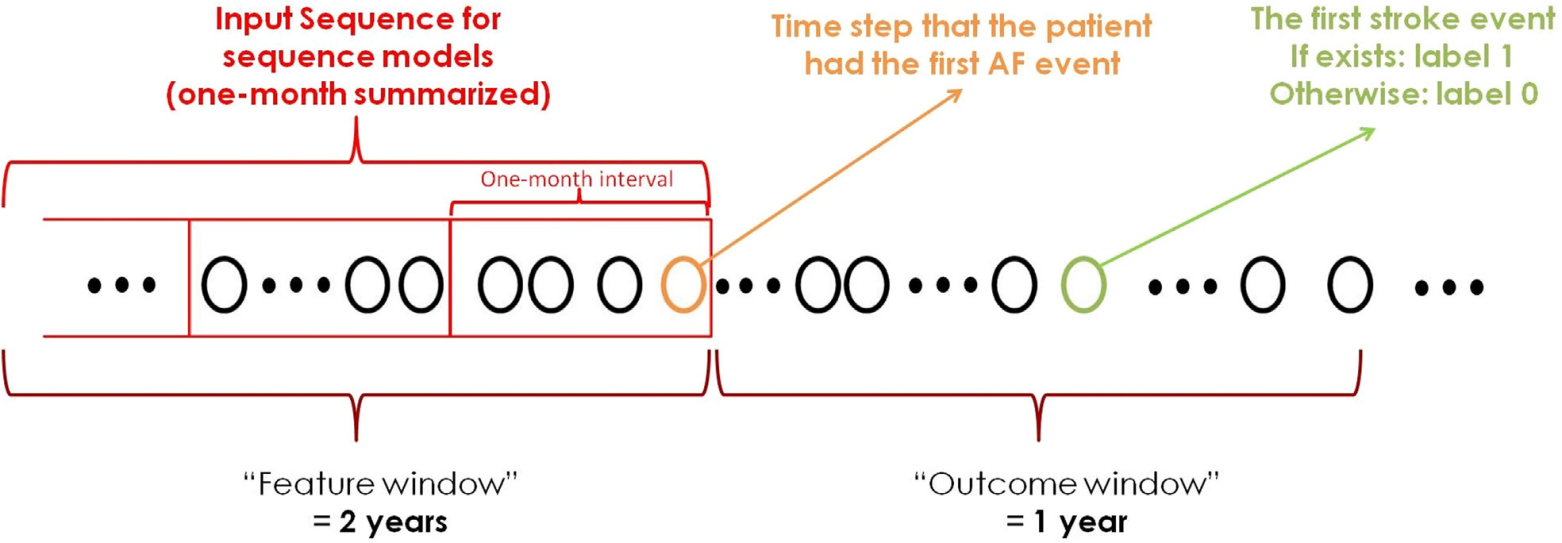
Study Design of Prediction Framework This figure illustrates the definition of our model input and prediction targets. Our model uses a patient’s electronic health records 2 years before the first atrial fibrillation as the input sequence. It predicts whether the patient will develop the first stroke within 1 year after the first atrial fibrillation, formulated as a binary classification. The input sequence is further summarized over 1 month to help the training process, which will be detailed in the Data Preprocessing section.

Figure 1 shows the study design, which includes a feature window and an outcome window. Each patient in the data set is represented by multiple NTUH-visiting records, each containing 40 features with missing data. We constructed input sequences and target labels for each patient using the timing of their first AF event. The raw input sequence comprised data from 2 years before the first AF occurrence. We used summarization techniques to preliminarily reduce the missing rate, unify time differences between data points, and control the total sequence length of the model’s input. To apply summarization, we first divide the 2-year period into 24 1-month intervals. Feature means for numerical features, and feature modes for categorical features were used as representative values for each 1-month timeframe. This resulted in a new input sequence of 24 summarized 40-dimensional records for each patient. We implemented 2 strategies for dealing with the remaining missing data: imputation using feature means and modes from the training set or replacing missing values with a unique sign (−1, imputation-free). The latter would be our primary setting, which aims to help the models learn better by clearly distinguishing missing values and depends on no prior statistical assumption. The statistics and missing rate of time-series features after summarization are shown in Supplemental Table 1. The outcome window focused on analyzing data within 1 year of the patient's initial AF occurrence to determine stroke risk classification.

### Dataset and decision threshold

The active cohort was split randomly into a training set (60%) and an independent test set (40%) with label stratification to maintain balanced class proportions. The independent test set was solely for testing and not involved in tuning or training. A validation set, comprising 20% of the training set, was used for adjusting model hyperparameters, with full details provided in the Supplemental Appendix. A down-sampling approach addressed the unbalanced label distribution in the data set, achieving a 4:1 ratio in the training set. Early stopping in deep learning models prevented overfitting by monitoring the validation set's loss across epochs. Model performance was evaluated on the independent test set using 1,000 bootstrap sets to calculate the 95% CI. A 3-fold validation was performed to assess the model's stability and generalization ability, provided in the Supplemental Appendix. Model outputs represented a risk score, ie, an uncalibrated probability of experiencing a stroke within 1 year after AF onset. As stroke risk prediction was modeled as binary classification, determining a patient classification threshold was necessary. Patients with risk scores surpassing the threshold were classified as high-risk (labeled 1) and those below as low-risk (labeled 0). Three settings were established and tested to examine models’ sensitivity to decision thresholds: the default (0.5), the threshold maximizing the Youden index, and the threshold maximizing the F1 score on the validation set. The latter 2 correspond to our primary threshold-sensitive evaluation metrics, which will be detailed in the Results section.

### Model building and explainable interpretability

We selected 4 classic models for nonsequential machine learning benchmarks: logistic regression (LR), support vector machine, random forest (RF), and extreme gradient boosting (XGB).^25^ The default input type for nonsequential benchmarks was the last summarization interval, including the first AF event. Additionally, we tested the effect of using the whole and flattened input sequence to assess the impact of employing multivariate time series with nonsequential benchmarks. For sequential machine learning benchmarking, we chose the gated recurrent unit (GRU), a successful version of the RNN with several gates to regulate time series encoding.^26^ The Supplemental Appendix lists the hyperparameters used for nonsequential benchmarks and GRU.

**Figure 2:**
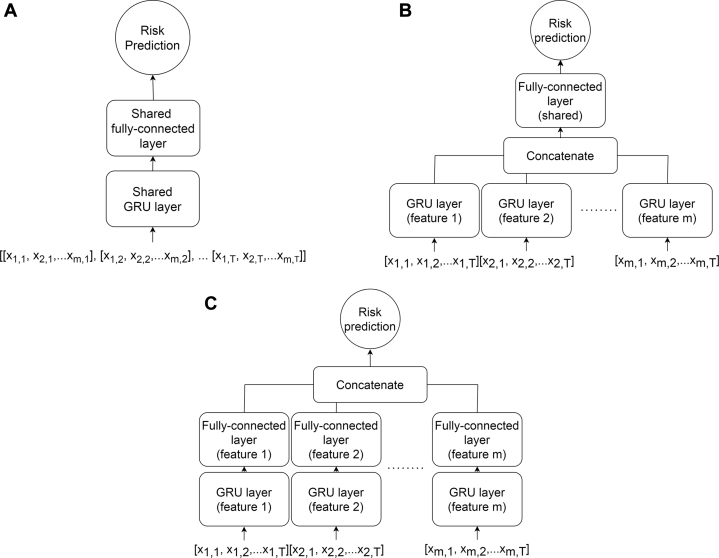
Model Architectures for Time-Series Prediction The schematic assumes a sequential data set with T time steps and m features per time step. (A) Standard gated recurrent unit (GRU), which uses the shared GRU and fully connected layer for all features to make predictions. (B) Forecasting Strokes via Interpretable Independent Networks (ForeSIIN) with a shared fully connected layer, which allows independent GRU layers for every feature. (C) The fully-independent structure of ForeSIIN with independent GRU and fully connected layers. Each model branch consisting of a distinct GRU and fully connected layer for a specific feature can be regarded as a sub-classifier and contribute to the final prediction independently.

For stroke risk prediction, we introduced the ForeSIIN model, with the GRU cell serving as its core architecture. Figures 2A illustrate a typical GRU cell and the prediction process of a conventional GRU. In ForeSIIN, each feature enters the GRU cell separately, allowing the model to generate distinct GRUs for extracting information from each input feature. This approach effectively blocks noise between features and preserves information to mitigate the impact of missing values. The encoded information from each feature is then passed through a fully connected (FC) layer, which can be shared (Figure 2B) or independent (Figure 2C). In this work, we adopt the latter since it is more interpretable. As shown in Figure 2C, each model branch, comprising a feature's GRU and FC layer, operates as a sub-classifier. These sub-classifiers communicate weakly through the composite loss to minimize noisy interactions between irrelevant and useful variables. Additionally, ForeSIIN can determine the contribution of each feature to the final risk estimate by calculating the total of the FC layer's outputs multiplied by the edge weights of the last neuron before the sigmoidal activation function. Positive feature contributions increase the output risk score, whereas negative contributions decrease it, defining the total of a feature as its perceptual feature effect.^27^

### Statistical analysis

Categorical variables were presented as counts with percentages, and continuous variables were expressed as mean ± SD. The chi-square test was used for comparisons between categorical variables, and the Student’s *t*-test was employed to compare continuous variables between 2 groups. Model performance was evaluated using various metrics, including the area under the precision-recall curve (AUPRC), the area under the receiver-operating characteristics curve (AUROC), and the concordance index (c-index), to assess model accuracy and to calculate CIs. The Kaplan-Meier estimator and log-rank test were used to analyze survival data.

Statistical analyses were conducted using Python (version 3.10) with machine learning libraries, including Scikit-learn and PyTorch, R (version 4.1.2, University of Auckland), and SPSS (version 25.0, SPSS Inc). A 2-tailed *P* value <0.05 was considered statistically significant.

## Results

### Clinical characteristics

The cohort research included a total of 7,710 AF patients in NTUH. The medium follow-up period was 22.3 months (Q1-Q3: 3.33-52.5 months). During the period from 2014 to 2019, a total of 788 of 7,710 AF patients experienced a TIA/IS. The annual TIA/IS incidence rate ranged from 181.96 (95% CI: 164.42-200.93) to 15.81 (95% CI: 12.38-20.18) per 1,000 person-years, with an overall incidence of 42.40 (95% CI: 39.60-45.39).

The training set and test set consisted of 4,626 and 3,084 individuals, respectively. The validation sample further split from the training set included 926 individuals with AF. Table 1 summarizes the clinical characteristics of individuals with AF. The mean age of the 2 split data sets was 73.9 ± 10.2 and 74.2 ± 10.1 years. The underlying diseases and echocardiographic investigations were comparable.

**Table 1:** The Baseline Characteristics of Patients With Atrial Fibrillation in NTUH

	Training Set (n = 4,626)	Test Set (n = 3,084)	*P* Value
Age, y	73.9 ± 10.2	74.2 ± 10.1	0.362
Male	2,641 (57.1)	1,732 (56.2)	0.420
BMI, kg/m^2^	25.3 ± 4.3	25.4 ± 4.3	0.622
Comorbidity			
HTN	1,866 (40.3)	1,189 (38.6)	0.122
DM	3,814 (82.4)	2,579 (83.6)	0.184
CAD	950 (20.5)	639 (20.7)	0.863
PAOD	361 (7.8)	260 (8.4)	0.326
HF hospitalization	298 (6.4)	211 (6.8)	0.512
History of TIA/stroke	257 (5.6)	165 (5.4)	0.721
Laboratory			
FPG, mg/dL	135.3 ± 68.7	137.1 ± 88.1	0.351
HbA1c, %	6.9 ± 1.4	6.0 ± 1.4	0.658
TCHO, mg/dL	161.6 ± 45.1	162.6 ± 45.1	0.717
TG, mg/dL	139.3 ± 103.3	141.8 ± 98.9	0.336
LDL, mg/dL	94.9 ± 32.7	94.4 ± 30.4	0.527
HDL, mg/dL	42.8 ± 13.1	42.4 ± 12.6	0.215
ALT, U/L	23.9 ± 32.1	23.4 ± 33.5	0.594
eGFR, mL/min/1.73 m^2^	64.8 ± 32.2	63.9 ± 32.4	0.347
Risk score			
CHA_2_DS_2_-VASc score	3.4 ± 1.6	3.4 ± 1.6	0.479
ATRIA	5.7 ± 2.6	5.8 ± 2.7	0.254
Echocardiography			
LA size, cm	4.3 ± 0.8	4.3 ± 0.9	0.877
DT, s	0.2 ± 0.1	0.2 ± 0.1	0.860
E, mm/s	96.3 ± 35.6	96.3 ± 34.6	0.923
A, mm/s	90.3 ± 29.9	91.8 ± 31.0	0.158
E/A, mm/s	1.4 ± 8.9	1.2 ± 4.6	0.387
LVEF, %	62.0 ± 13.5	62.1 ± 13.7	0.856
LVIDs, cm	3.2 ± 0.8	3.2 ± 0.8	0.736
LVIDd, cm	4.8 ± 0.7	4.8 ± 0.7	0.626
LV mass, gm	212.5 ± 68.8	210.5 ± 67.6	0.273
TR Vmax, cm/s	31.7 ± 13.1	31.5 ± 12.5	0.569
TIA/ischemic stroke 1 y	400 (8.6)	272 (8.8)	0.805

A = late diastolic mitral annular velocity; ALT = alanine aminotransferase; BMI = body mass index; CAD = coronary artery disease; DM = diabetes mellitus; DT = deceleration time; E = early diastolic mitral annular velocity; eGFR = estimated glomerular filtration rate; FPG = fasting plasma glucose; HbA1c = glycosylated hemoglobin; HDL = high-density lipoprotein; HF = heart failure; HTN = hypertension; LA = left atrial size; LDL = low-density lipoprotein; LV = left ventricle; LVIDd = left ventricular internal diameter end diastole; LVIDs = left ventricular internal diameter end systole; PAOD = peripheral arterial occlusive disease; TCHO = total cholesterol; TG = triglyceride; TIA = transient ischemic attack; TR Vmax = peak velocity of tricuspid regurgitation.

### Model performance

Table 2 presents the performance comparison of the independent test set, evaluating 2 imputation settings (with feature mean/mode or substituted with −1) for each model and 2 input types (final summarization interval or the whole input sequence) for nonsequential benchmarks. The ForeSIIN model achieved the best performance, with an AUPRC of 0.210 (95% CI: 0.137-0.283), 47% higher than the highest statistical score (ATRIA: 0.143; 95% CI: 0.073-0.201) and 24% better than the second-best model (RF: 0.169; 95% CI: 0.095-0.230). Additionally, the ForeSIIN model attained an AUROC of 0.764 (95% CI: 0.722-0.810), 9% higher than the best performance achieved by the statistical model (ATRIA: 0.703; 95% CI: 0.657-0.754) and 6% higher than the second-best model (XGB: 0.722; 95% CI: 0.676-0.769).

**Table 2:** Model Performance for Ischemic Stroke Risk Within 1 Year

	AUPRC (95% CI)	AUROC (95% CI)	Youden Index (95% CI)	F1 Score (95% CI)	Harrel’s C-Statistics (95% CI)
CHA_2_DS_2_-VASc score	0.103 (0.040-0.142)	0.650 (0.596-0.699)	0.223 (0.132-0.302)	0.112 (0.041-0.181)	0.645 (0.598-0.695)
ATRIA score	0.143 (0.073-0.201)	0.703 (0.657-0.754)	0.262 (0.166-0.356)	0.134 (0.056-0.204)	0.695 (0.647-0.746)
LR^a^^,^^b^	0.047 (0.035-0.059)	0.580 (0.529-0.633)	0.117 (0.035-0.205)	0.000 (0.000-0.000)	0.584 (0.536-0.634)
SVM^c^^,^^d^	0.153 (0.083-0.210)	0.691 (0.637-0.741)	0.260 (0.171-0.351)	0.251 (0.175-0.328)	0.686 (0.635-0.733)
RF^a^^,^^d^	0.169 (0.095-0.230)	0.689 (0.637-0.742)	0.253 (0.171-0.337)	0.262 (0.188-0.336)	0.677 (0.624-0.731)
XGB^c^^,^^d^	0.144 (0.077-0.196)	0.722 (0.676-0.769)	0.311 (0.221-0.399)	0.190 (0.111-0.260)	0.713 (0.670-0.759)
GRU^b^^,^^c^	0.071 (0.045-0.091)	0.633 (0.581-0.691)	0.160 (0.067-0.253)	0.144 (0.102-0.183)	0.644 (0.591-0.698)
ForeSIIN^b^^,^^c^	**0.210 (0.137-0.283)**	**0.764 (0.722-0.810)**	**0.373 (0.279-0.463)**	**0.280 (0.209-0.356)**	**0.758 (0.717-0.802)**

AUPRC = area under the precision-recall curve; AUROC = area under the receiver-operating characteristics curve; ForeSIIN = Forecasting Strokes via Interpretable Independent Networks; GRU = gated recurrent units; LR = logistic regression; RF = random forest; SVM = support vector machine; XGB = XGBoost.

a Mean/mode imputation.

b Input the complete input sequence (flattened in the nonsequential model case).

c No imputation (replace with −1).

d Only input the last summarization interval containing the first atrial fibrillation event.

**Figure 3:**
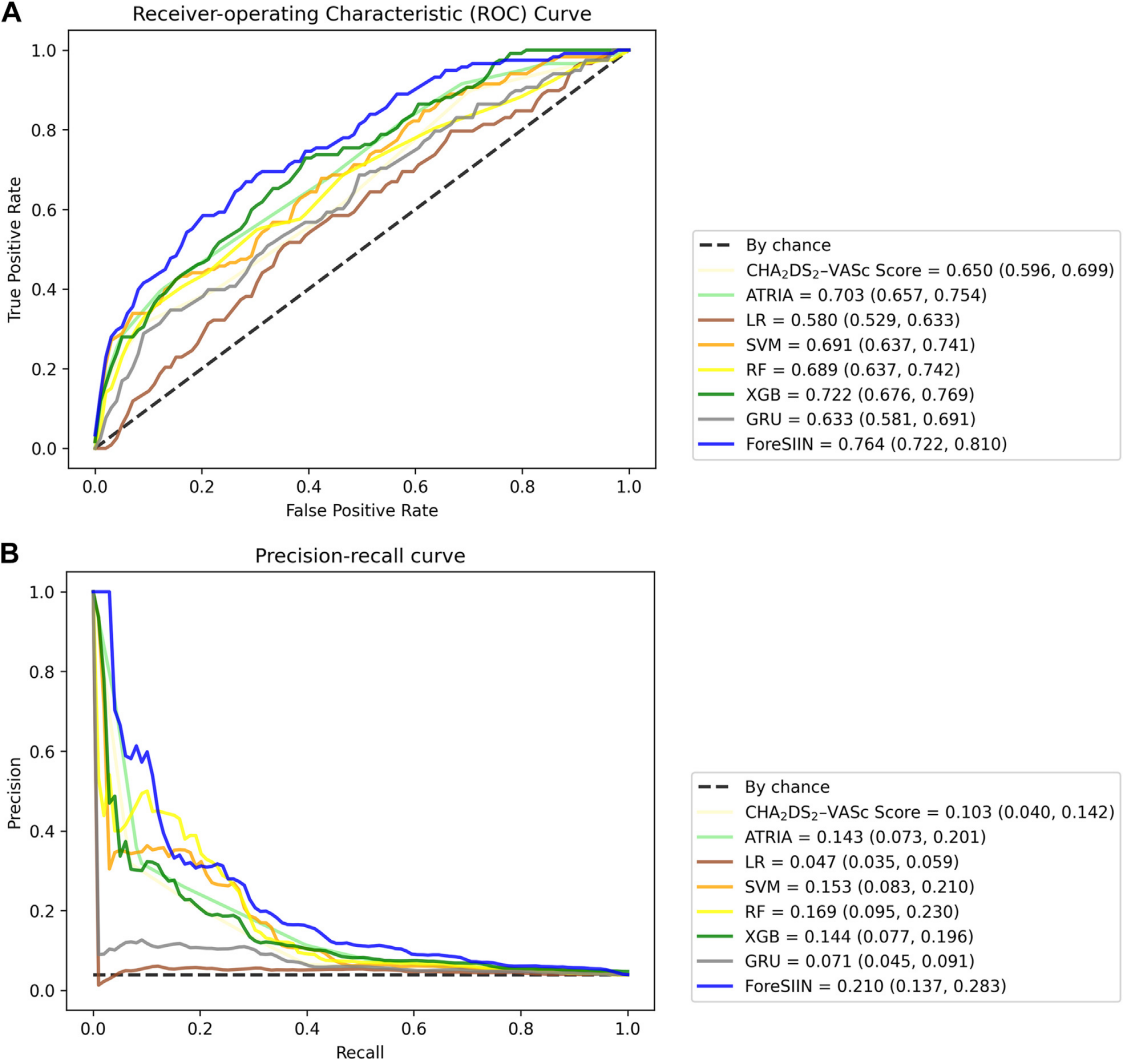
Model Performance Metrics This figure shows the curves comparing predictive performance across models. The tables on the lower right list the area under curves with the lower/upper bounds of the 95% CI. (A) Receiver-operating characteristic (ROC) curves. (B) Precision-recall curves (PRC). LR = logistic regression; RF = random Forest; SVM = support vector machine; XGB = extreme gradient boosting; other abbreviations as in Figure 2.

Moreover, the Youden index and F1 score were used to assess the actual classification ability. The ForeSIIN model achieved a Youden index of 0.373 (95% CI: 0.279-0.463), 42% higher than the highest statistical score (ATRIA: 0.262; 95% CI: 0.166-0.356) and 20% better than the second-best model (XGB: 0.311; 95% CI: 0.221-0.399). Additionally, the ForeSIIN model derived an F1 score of 0.280 (95% CI: 0.209-0.356), 109% higher than the highest statistical score (ATRIA: 0.134; 95% CI: 0.056-0.204) and 7% better than the second-best model (RF: 0.262; 95% CI: 0.188-0.336). ROC and PRC curves for various methods are illustrated in Figure 3. The hyperparameters for models and detailed performance comparison are shown in Supplemental Tables 2 and 3. Supplemental Tables 4 and 5 contain extra experiments of ForeSIIN’s efficacy under different feature sets and model structures. Supplemental Table 6 shows the results of the 3-fold validation of the ForeSIIN model. Overall, ForeSIIN still outperformed the benchmarks in the additional experiments. The external validation on an independent cohort was collected by the NTUH Yunlin Branch with a sample size of 6,822 AF patients. The detailed results of external validation are listed in Supplemental Table 7. In the external validation, ForeSIIN demonstrated noninferior predictive performance with an AUROC of 0.646 (95% CI: 0.618-0.671) and the best AUPRC of 0.222 (95% CI: 0.184-0.259). We also conducted the decision curve analysis (DCA) to examine the potential clinical impact (Supplemental Figure 1).

### Feature impact analysis

**Figure 4:**
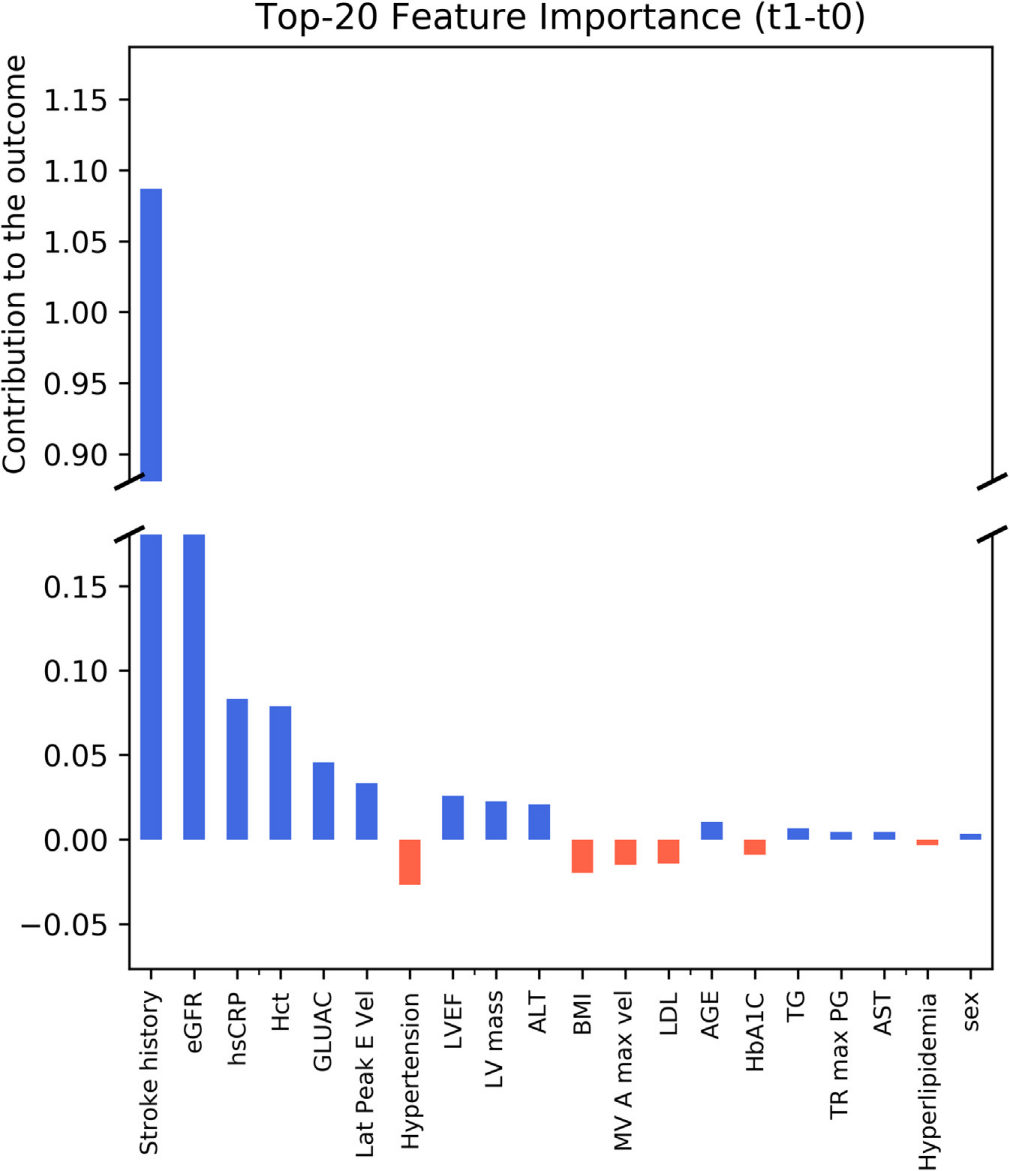
Feature Impact Analysis Feature contributions to stroke prediction were quantified using their influence on the model’s logit output in the ForeSIIN framework. The 20 most impactful features are presented, ranked by the average difference between patients who did and did not experience ischemic stroke within 1 year of atrial fibrillation (AF) onset (“t1–t0”). Positive values reflect stronger predictive separation. ALT = alanine aminotransferase; AST = aspartate transferase; BMI = body mass index; eGFR = estimated glomerular filtration rate; GLUAC = fasting glucose; HbA1c = glycosylated hemoglobin; Hct = hematocrit; hsCRP = high-sensitivity C-reactive protein; LDL = low-density lipoprotein; LVEF = left ventricular ejection fraction; TG = triglyceride; TR max PG = peak tricuspid regurgitation velocity.

For the feature impacts reported by the ForeSIIN model, we first define 3 sets of results on the independent test set: the average feature impacts of patients without a stroke within 1 year from their first AF event (group t0), patients who had a stroke within 1 year from their first AF event (group t1), and their feature impact difference (t1 − t0). We focus on the t1 − t0 result because it provides the most significant medical insight by reflecting the distinguishable feature impacts between patients with/without stroke. Benefitting from ForSIIN’s independent network design for each input feature, we can intuitively calculate their individual contributions to the model’s output (predicted risk score). This allows us to observe how ForSIIN’s predictions are made, enhancing its interpretability. Figure 4 shows the contributions of the top 20 features, ordered by their absolute quantities. The larger (more positive) an input feature’s value in the difference (t1 − t0) calculation is, the more contribution it has made to separate the patients with/without a stroke in 1 year and otherwise. The history of TIA/IS plays a dominant role, along with essential features, including eGFR, hsCRP, and hematocrit. Detailed feature impact values and more investigations on the feature impact are listed in the appendix (Supplemental Figure 2, Supplemental Tables 8 to 11).

### Survival analysis

**Figure 5:**
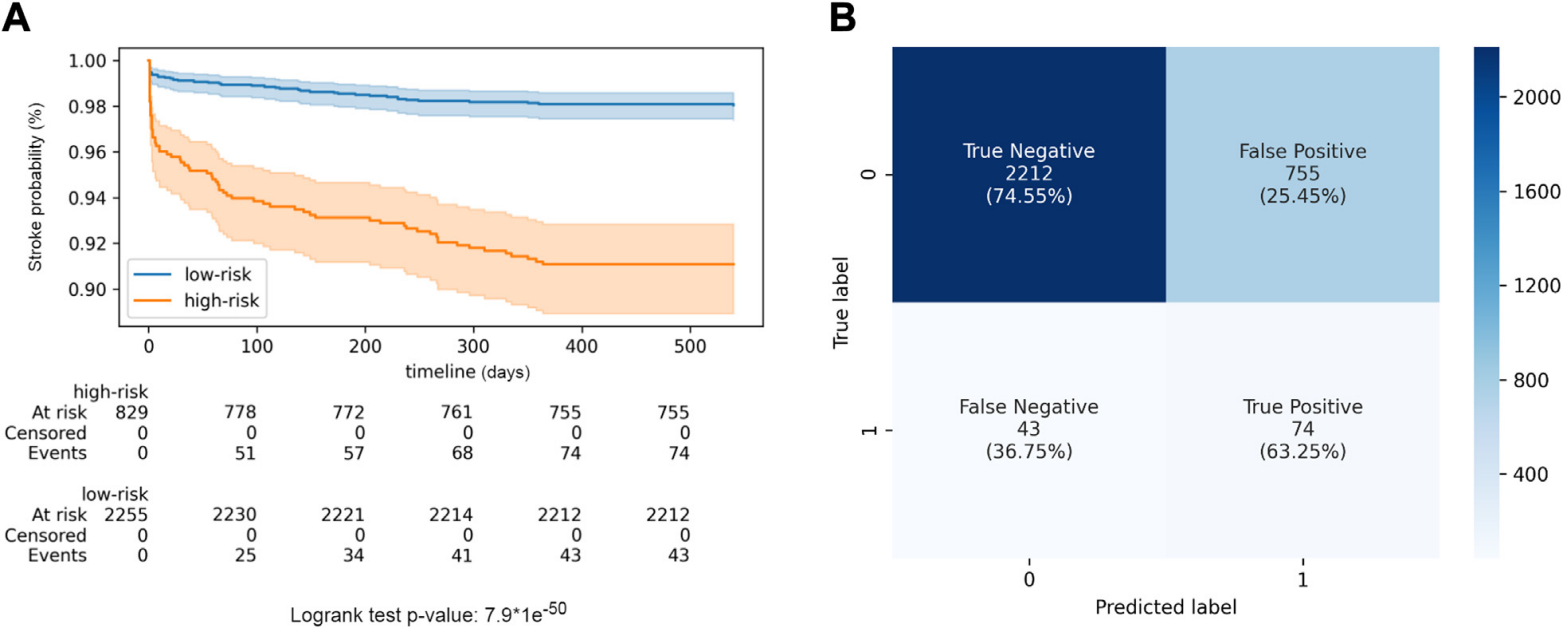
Kaplan-Meier Analysis and Confusion Matrix (A) Kaplan-Meier curve stratified by predicted stroke risk using the decision threshold maximizing the Youden index on the validation set. (B) Confusion matrix at the same threshold. Parenthetical values indicate true negative rate, false positive rate, false negative rate, and true positive rate. Recall, precision, and geometric mean (G-mean) were 0.63, 0.09, and 0.69, respectively.

Figure 5A demonstrates the result with the threshold maximizing the Youden index, effectively classifying patients into low/high-risk groups with a *P* value <0.05. In Figure 5B, we show distinct sensitivity, specificity, and precision trade-offs with the same Youden-based threshold. Although our model primarily focuses on labeling 1-year stroke after initial AF episodes, Kaplan-Meier analysis provides additional insights into stroke development in AF patients classified as high- or low-risk. The analyses for other thresholds are listed in Supplemental Figure 3.

## Discussion

In this study, we developed ForeSIIN, a deep-learning stratification system that leverages temporal data through a novel recurrent independent network architecture. This imputation-free model provides precise stroke risk predictions for AF patients, addressing the limitations of traditional methods. By employing a high-dimensional time series-integrated RNN, ForeSIIN captures the intricate relationships among AF, IS, and evolving patient features, enhancing both interpretability and predictive accuracy (Central Illustration). Compared with conventional risk scores, ForeSIIN showed remarkable improvements, outperforming the ATRIA score by 47% and the CHA_2_DS_2_-VASc score by 104% in AUPRC. To our knowledge, this represents the first and largest study to apply RNNs to stroke prediction in AF patients.

**Central Illustration:**
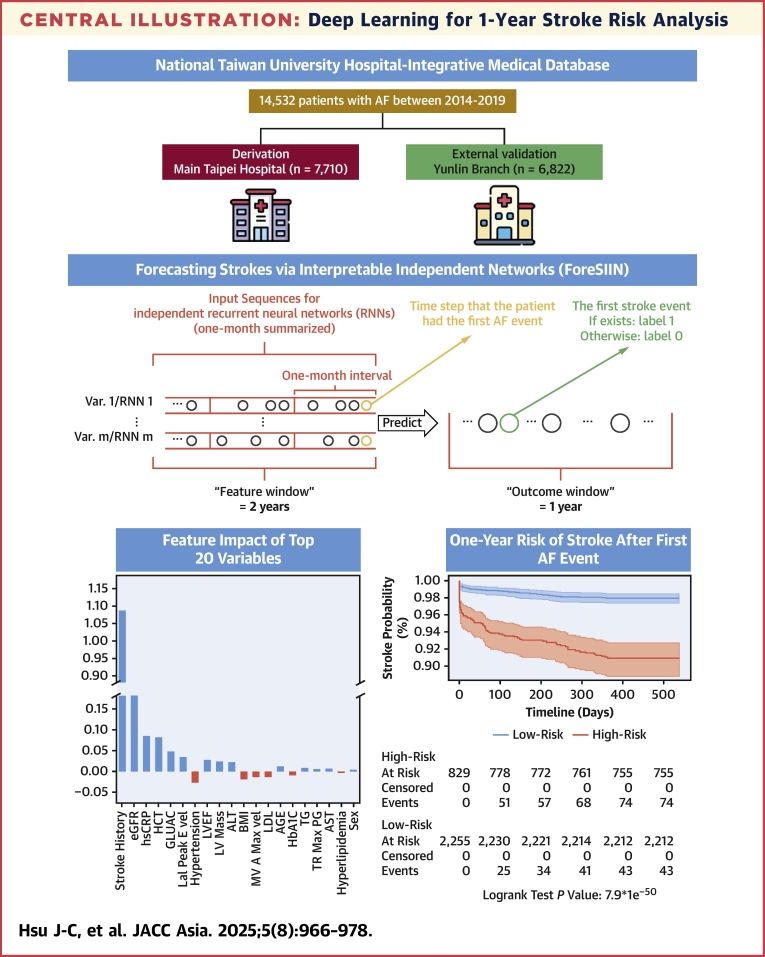
Deep Learning for 1-Year Stroke Risk Analysis A schematic of Forecasting Strokes via Interpretable Independent Networks (ForeSIIN), a novel deep learning framework leveraging gated recurrent units (GRUs) to predict 1-year ischemic stroke risk in patients with atrial fibrillation (AF). The model captures temporal feature importance and provides interpretable predictions through feature-specific sequential modeling.

The CHA_2_DS_2_-VASc score remains the cornerstone for stroke risk stratification in AF,^28^ but its reliance on baseline characteristics fails to capture the evolving nature of comorbidities, which can markedly influence stroke risk over time. The integration of dynamic clinical, laboratory, imaging, and ethnic data into predictive models could enhance the accuracy of stroke risk prediction and inform more nuanced anticoagulation decisions. Recent advances in stroke risk prediction for AF patients have increasingly incorporated ML models.^29^ Although traditional methods, such as logistic and stepwise regression, have demonstrated limited predictive accuracy (c-index ranging from 0.65 to 0.71), newer techniques, including deep neural networks and ensemble learning methods, have shown improved performance.^30^^,^^31^ In contrast to models that rely solely on baseline data, our dynamic approach accounts for time-varying comorbidities, allowing for real-time stroke risk prediction and enhancing both predictive accuracy and clinical applicability.^32^ This temporal dimension enables the model to better capture the evolving nature of stroke risk factors in AF patients.^33^ Furthermore, the identification of key predictors such as age, chronic kidney disease, and history of TIA/IS is consistent with previous studies, reinforcing their critical role in stroke risk stratification. The substantially larger sample size in our study further bolsters the statistical power and generalizability of our findings.^34^ The model’s robust performance across both internal and external validation cohorts highlights its potential for broader clinical application, particularly in the Asian AF population.

Our study did not limit the AF population to anticoagulant-naïve individuals but instead followed contemporary AF guidelines for anticoagulant use.^35^ The incidence rate of TIA/IS has significantly decreased from 179.31 per 1,000 person-years in 2014 to 15.54 per 1,000 person-years in 2019 (with annual rates of 179.31, 63.17, 34.01, 21.99, 19.35, and 15.54, respectively). This decline may reflect improvements in treatment, prevention strategies, and other contributing factors over the years. In this study, we identified a history of TIA/IS as the strongest predictor of future stroke risk, while factors such as eGFR, hsCRP emerged as significant contributors. Consistent with previous findings, our results highlight that patients with AF remain at significant residual risk for IS and other complications despite anticoagulation therapy. Prior research has identified key risk factors, including a history of thromboembolism, advanced age, persistent AF, diabetes, chronic obstructive pulmonary disease, and the absence of antiarrhythmic therapy.^36^^,^^37^ Our model aligns with these findings, reinforcing the need for comprehensive risk stratification in this high-risk population. Reduced renal function may heighten stroke risk through procoagulant and inflammatory mechanisms, contributing to atherosclerosis and thrombosis.^38^ Associations among renal failure, atrial cardiomyopathy, cardiac dysfunction, and stroke risk have been explored.^39^ Age, serum glycosylated hemoglobin, fasting glucose levels, left ventricular ejection function, LVM, and diastolic function also significantly impact stroke prediction. Integrating echocardiographic measurements with CHADS_2_ score or CHA_2_DS_2_-VASc score may enhance stroke risk identification in AF patients.^40^ The ForeSIIN model enhances interpretability by conveying learned insights, fostering an understanding of its predictive mechanisms.

The relationship between AF and IS extends beyond cardioembolic strokes, warranting investigation into their interconnected pathophysiological mechanisms.^41^, ^42^, ^43^ Atrial dysfunction in AF can induce a proinflammatory state and oxidative stress, exacerbating vascular homeostasis imbalance.^44^ The bidirectional association between AF and IS suggests that stroke may precede AF diagnosis by years.^45^ Given overlapping risk factors, proactive AF management and extensive AF screening in stroke patients are imperative. Long-term monitoring and implanted cardiac monitors facilitate AF diagnosis and anticoagulant initiation, reducing recurrent stroke risk.

Our ForeSIIN model effectively differentiates between low- and high-stroke risk groups within 1 year, based on both the number and severity of characteristic features. This approach not only enhances predictive performance but also provides a novel perspective on patient care by enabling closer monitoring and personalized follow-up. Future applications may involve tracking the escalation of stroke risk as comorbidities evolve, leveraging this dynamic approach for more timely interventions.

### Study limitations

First, the retrospective design of the analysis restricts the ability to establish causal relationships. Nonetheless, the large sample size and the continuous monitoring of patients allowed for the integration of RNN with EHR data, thereby enhancing the reliability and robustness of our findings. Second, although we adjusted for a number of potential confounders, certain variables were not included in the analysis. We did not account for the subtypes or burden of AF, nor did we consider lifestyle factors such as alcohol consumption, sleep apnea, or adherence to anticoagulant therapy. The exclusion of these factors may introduce selection bias, potentially limiting the generalizability of our model. Finally, the study was conducted within an East Asian cohort, which may constrain the applicability of the results to other ethnic populations. Future studies should seek to externally validate this model in more diverse and heterogeneous populations to determine its broader applicability and generalizability.

## Conclusions

The innovative ForeSIIN model demonstrated accurate stroke prediction in patients with AF and enhanced interpretation of dynamic information when AF coexists with several comorbidities across time.

### Availability of Data and Materials

The data sets used in this study are available only at the National Taiwan University Hospital. Data is available on request caused by ethical restrictions.

## Funding Support and Author Disclosures

This work is supported by grants from the Ministry of Science and Technology Council of Taiwan (MOST 110-2221-E-002-112-MY3 and 113-2222-E-002-008), the Ministry of Education (113M7054), and the Ministry of Health and Welfare (MOHW 111-TDU-B-221-114003, MOHW 113-TDU-B-221-134003, MOHW 114-TDU-B-221-144003). This work was financially supported by the Center for Advanced Computing and Imaging in Biomedicine (NTU-114L900701) from The Featured Areas Research Center Program within the framework of the Higher Education Sprout Project by the Ministry of Education in Taiwan. The authors have reported that they have no relationships relevant to the contents of this paper to disclose.
