# Supplementary material for: Interpretable Independent Recurrent Networks for Forecasting Stroke in Atrial Fibrillation
Source: JACC Asia. 2025 Jun 10;5(8):966–78. doi: 10.1016/j.jacasi.2025.04.003 (PMC12426851; doi:10.1016/j.jacasi.2025.04.003)
Supplement: Supplemental Figures 1-3 and Supplemental Tables 1-11 [file mmc1.docx]

**Supplemental materials**

**Supplemental Figure 1. Decision curve analysis.**

We conducted the analysis for the main results shown in Table 2 of the main text.


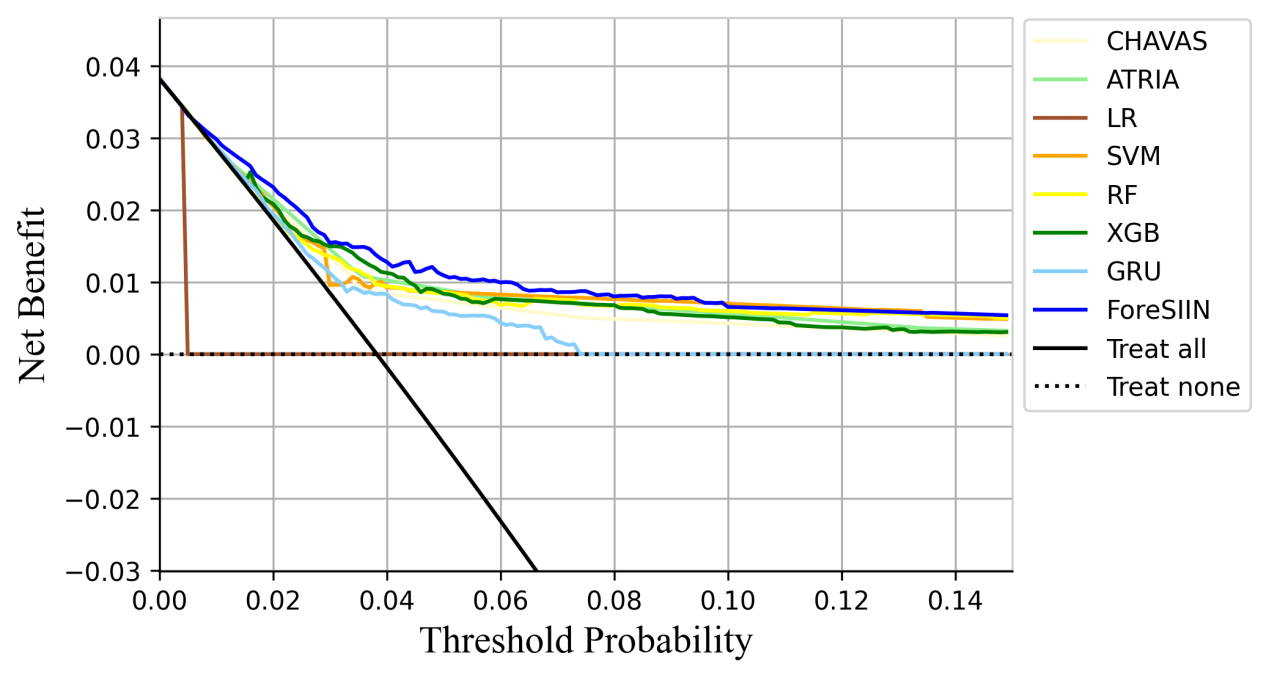


**Supplemental Figure 2. Feature impact analysis on more subgroups.** Feature impacts were evaluated by their contributions to the logit. Here, we show the top 20 impactful features. The detailed feature impacts are listed in Sup Table 8-11. The notation “t” indicates the true label (0 or 1), “p” indicates the predicted label (0 or 1) with a decision threshold maximizing the Youden statistic, and “h” indicates the presence of stroke history before the first AF event (0 or 1). (A) t0, (B) t1: the average feature impacts of the patients who will/will not undergo a stroke in one year. (C) t0p0, (D) t1p1: to observe the feature impacts of correctly identified low/high-risk patients (E) t0p0h1: to observe the feature impacts of correctly identified low-risk patients with a dominant high-risk factor (F) t1p1h0: to observe the feature impacts of correctly identified high-risk patients with a dominant low-risk factor.


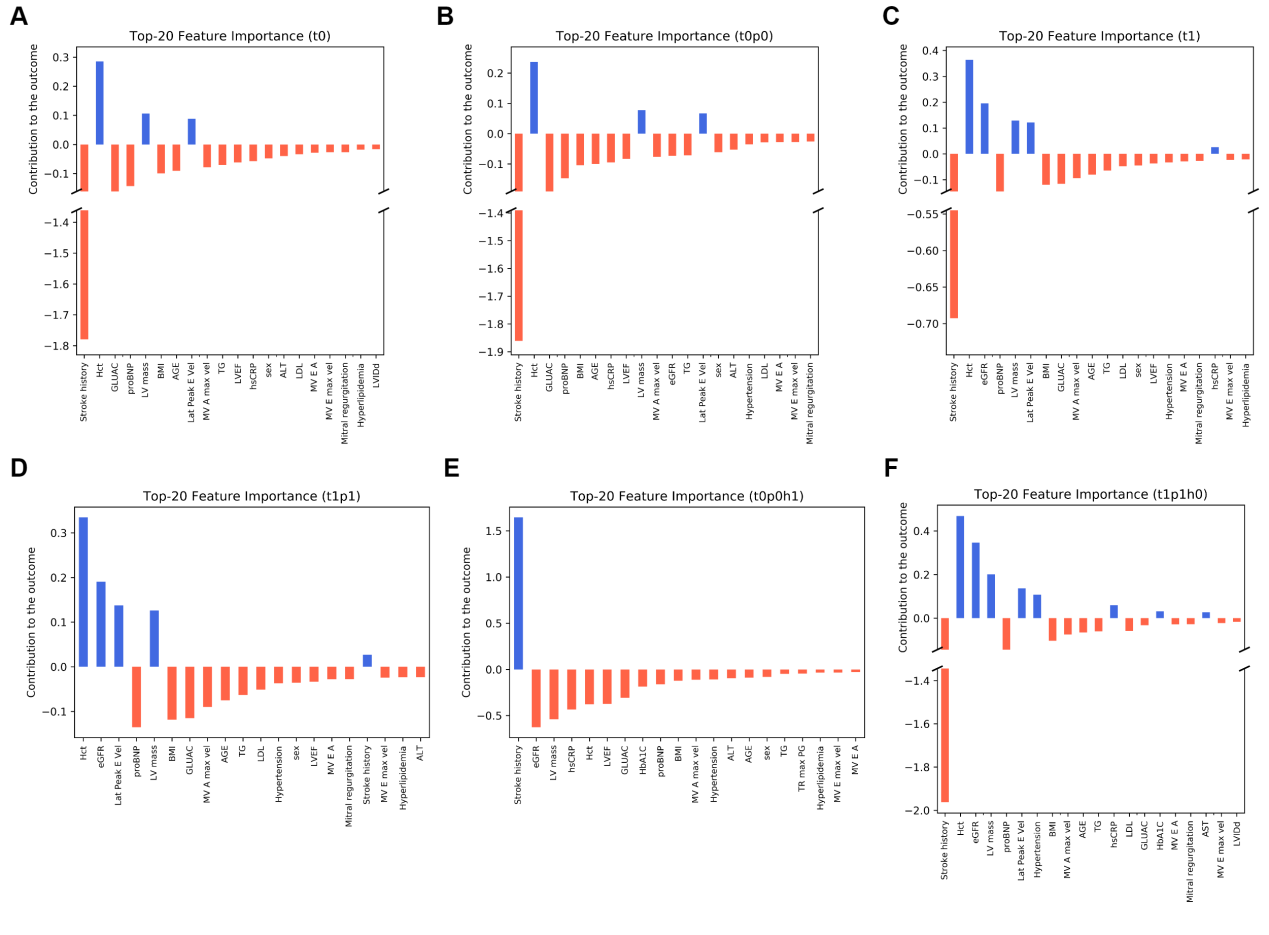


**Supplemental Figure 3. Kaplan-Meier analyses and confusion matrices with more decision thresholds**

(A) and (C): Kaplan-Meier plot and confusion matrix with the default decision threshold (0.5). (B) and (D): Kaplan-Meier plot and confusion matrix with the decision threshold maximizing the F1-score on the validation set. The numbers in the parentheses of the confusion matrix denote the corresponding true negative rate (TNR), false positive rate (FPR), false negative rate (FNR), and true positive rate (TPR).


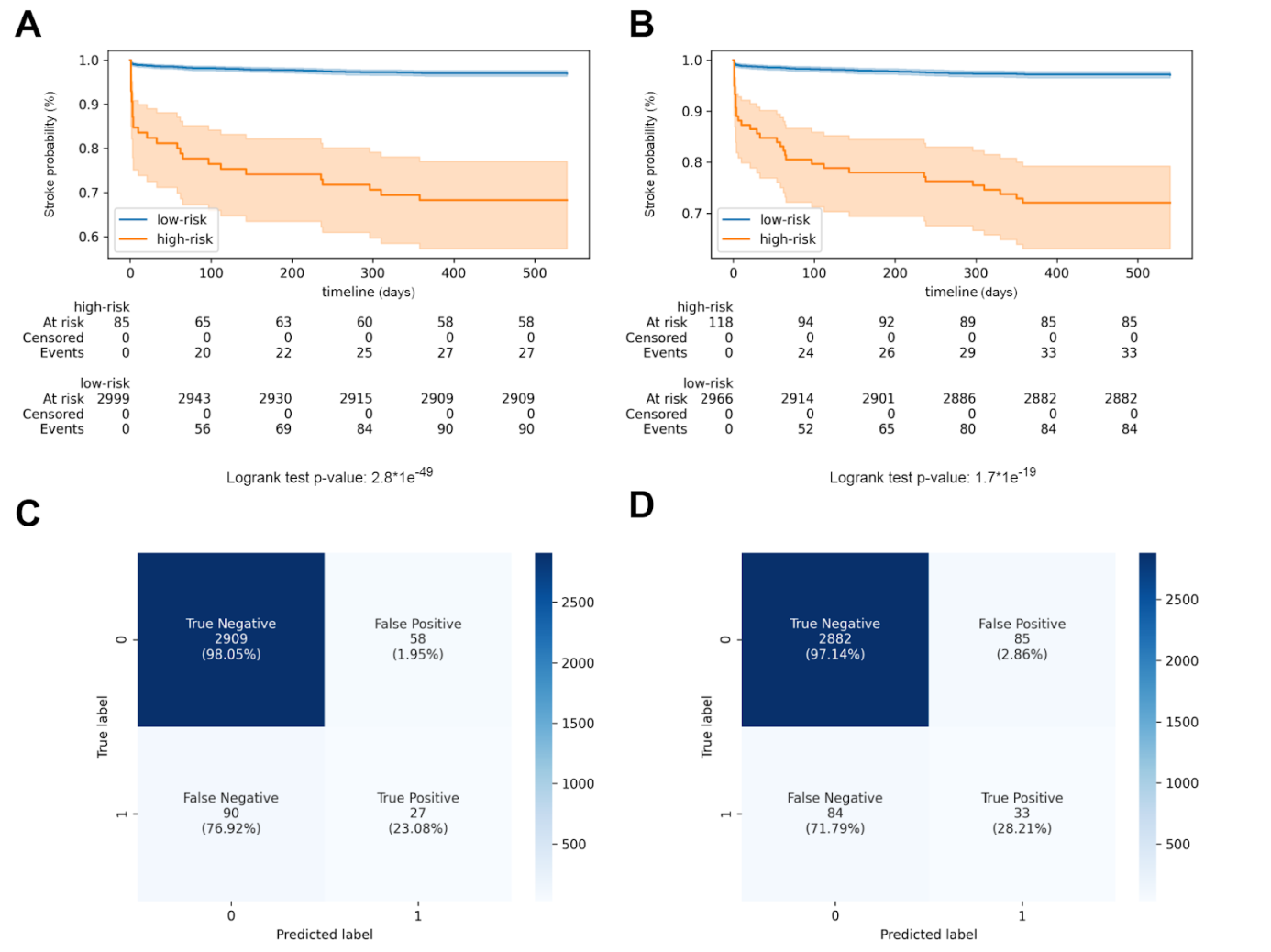


**Supplemental Table 1.** The statistics and missing rate of time-series features after summarization

|  | Training set | | | Validation set | | | Test set | | |
| --- | --- | --- | --- | --- | --- | --- | --- | --- | --- |
|  | Oldest observation before present  (unit: summarization intervals) | Number of summarization intervals with observations | Missing rate | Oldest observation before present  (unit: summarization intervals) | Number of summarization intervals with observations | Missing rate | Oldest observation before present  (unit: summarization intervals) | Number of summarization intervals with observations | Missing rate |
| Age | 12.6±8.8 | 10.1±8.1 | -- | 12.5±8.7 | 10.2±8.1 | -- | 12.2±8.4 | 10.1±8.0 | -- |
| HbA1C | 5.4±1.7 | 8.6±2.9 | 0.8 | 5.5±1.7 | 8.6±2.8 | 0.8 | 5.1±1.6 | 8.4±2.7 | 0.8 |
| Glucose | 5.8±1.9 | 8.8±3.4 | 0.8 | 5.8±1.9 | 8.8±3.4 | 0.8 | 5.5±1.8 | 8.6±3.4 | 0.8 |
| TG | 5.1±1.3 | 8.2±2.2 | 0.8 | 5.2±1.3 | 8.2±2.1 | 0.8 | 4.9±1.3 | 8.1±2.3 | 0.8 |
| TCHO | 4.8±1.2 | 8.0±2.1 | 0.8 | 4.7±1.2 | 7.9±2.0 | 0.8 | 4.6±1.1 | 7.9±2.1 | 0.8 |
| LDL | 4.2±1.1 | 7.7±2.0 | 0.8 | 4.4±1.1 | 7.8±2.0 | 0.8 | 4.0±1.0 | 7.5±2.0 | 0.9 |
| HDL | 3.6±0.8 | 7.1±1.6 | 0.9 | 3.4±0.8 | 7.0±1.6 | 0.8 | 3.4±0.8 | 7.0±1.7 | 0.9 |
| AST | 3.7±1.4 | 7.1±3.2 | 0.8 | 4.1±1.5 | 7.4±3.5 | 0.8 | 3.6±1.3 | 7.0±3.2 | 0.8 |
| ALT | 5.6±2.1 | 8.4±3.9 | 0.7 | 5.7±2.2 | 8.5±4.1 | 0.7 | 5.2±1.9 | 8.2±3.7 | 0.7 |
| eGFR | 3.2±1.3 | 6.8±3.2 | 0.8 | 3.4±1.5 | 6.9±3.5 | 0.8 | 2.9±1.2 | 6.4±3.1 | 0.8 |
| hsCRP | 1.7±0.6 | 4.7±1.6 | 0.8 | 1.8±0.6 | 4.8±1.5 | 0.8 | 1.6±0.6 | 4.6±1.5 | 0.8 |
| Hct | 5.3±2.4 | 8.1±4.4 | 0.6 | 5.5±2.5 | 8.1±4.5 | 0.6 | 5.1±2.3 | 8.0±4.3 | 0.6 |
| NT-proBNP | 0.4±0.1 | 2.1±0.5 | 0.9 | 0.4±0.1 | 2.1±0.5 | 0.9 | 0.4±0.1 | 2.2±0.6 | 0.9 |
| BNP | 0.3±0.1 | 2.0±0.3 | 0.97 | 0.3±0.1 | 1.8±0.3 | 0.97 | 0.2±0.1 | 1.7±0.3 | 0.97 |
| BMI | 11.4±6.8 | 10.2±7.1 | 0.4 | 11.4±6.9 | 10.2±7.0 | 0.4 | 11.0±6.5 | 10.1±6.8 | 0.4 |
| LVIDd | 2.1±0.4 | 5.1±0.8 | 0.8 | 2.2±0.4 | 5.1±0.7 | 0.8 | 2.0±0.4 | 5.0±0.7 | 0.8 |
| LVIDs | 2.1±0.4 | 5.1±0.8 | 0.8 | 2.2±0.4 | 5.1±0.7 | 0.8 | 2.0±0.4 | 5.0±0.7 | 0.8 |
| LV mass | 2.1±0.4 | 5.1±0.7 | 0.8 | 2.2±0.4 | 5.1±0.7 | 0.8 | 1.9±0.4 | 5.0± | 0.8 |
| LA size | 2.0±0.4 | 5.1±0.7 | 0.8 | 2.1±0.4 | 5.1±0.7 | 0.8 | 1.9±0.4 | 4.9±0.7 | 0.8 |
| E | 2.0±0.4 | 5.0±0.7 | 0.8 | 2.0±0.4 | 5.0±0.7 | 0.8 | 1.9±0.4 | 4.9±0.7 | 0.8 |
| A | 1.7±0.3 | 4.8±0.7 | 0.9 | 1.7±0.3 | 4.7±0.6 | 0.9 | 1.6±0.3 | 4.7±0.6 | 0.9 |
| E/A ratio | 1.7±0.3 | 4.8±0.7 | 0.9 | 1.7±0.3 | 4.7±0.6 | 0.9 | 1.6±0.3 | 4.7±0.6 | 0.9 |
| DT | 1.8±0.3 | 4.9±0.7 | 0.9 | 1.9±0.4 | 4.9±0.6 | 0.8 | 1.8±0.3 | 4.8±0.7 | 0.9 |
| TR max PG | 2.0±0.4 | 5.0±0.7 | 0.8 | 2.1±0.4 | 5.1±0.7 | 0.8 | 1.9±0.4 | 4.9±0.7 | 0.8 |
| E’ | 0.4±0.1 | 2.1±0.4 | 0.9 | 0.5±0.1 | 2.4±0.4 | 0.9 | 0.4±0.1 | 2.0±0.4 | 0.9 |
| A’ | 0.9±0.03 | 0.8±0.2 | 0.97 | 0.2±0.0 | 1.4±0.2 | 0.98 | 0.1±0.0 | 1.0±0.2 | 0.98 |
| LVEF | 2.1±0.4 | 5.1±0.8 | 0.8 | 2.2±0.4 | 5.1±0.7 | 0.8 | 2.0±0.4 | 5.0±0.7 | 0.8 |
| Hypertension history | 13.0±9.0 | 9.9±8.0 | 0.2 | 12.9±8.9 | 10.0±8.0 | 0.2 | 12.5±8.6 | 9.9±7.8 | 0.2 |
| Hyperlipidemia history | 13.0±9.0 | 9.9±8.0 | 0.2 | 12.9±8.9 | 10.0±8.0 | 0.2 | 12.5±8.6 | 9.9±7.8 | 0.2 |
| Gout history | 13.0±9.0 | 9.9±8.0 | 0.2 | 12.9±8.9 | 10.0±8.0 | 0.2 | 12.5±8.6 | 9.9±7.8 | 0.2 |
| COPD history | 13.0±9.0 | 9.9±8.0 | 0.2 | 12.9±8.9 | 10.0±8.0 | 0.2 | 12.5±8.6 | 9.9±7.8 | 0.2 |
| AMI history | 13.0±9.0 | 9.9±8.0 | 0.2 | 12.9±8.9 | 10.0±8.0 | 0.2 | 12.5±8.6 | 9.9±7.8 | 0.2 |
| PAOD history | 13.0±9.0 | 9.9±8.0 | 0.2 | 12.9±8.9 | 10.0±8.0 | 0.2 | 12.5±8.6 | 9.9±7.8 | 0.2 |
| Mitral regurgitation | 1.8±0.4 | 4.8±0.7 | 0.8 | 1.8±0.4 | 4.8±0.7 | 0.8 | 1.7±0.3 | 4.6±0.7 | 0.9 |
| Aortic regurgitation | 1.4±0.3 | 4.2±0.6 | 0.9 | 1.5±0.3 | 4.4±0.6 | 0.9 | 1.4±0.3 | 4.2±0.6 | 0.9 |
| Gender | 12.6±8.8 | 10.1±8.1 | -- | 12.5±8.7 | 10.2±8.1 | -- | 12.2±8.4 | 10.1±8.0 | -- |
| DM history | 12.6±8.8 | 10.1±8.1 | 0.3 | 12.5±8.7 | 10.2±8.1 | 0.3 | 12.2±8.4 | 10.1±8.0 | 0.3 |
| HF history | 13.0±9.0 | 9.9±8.0 | 0.2 | 12.9±8.9 | 10.0±8.0 | 0.2 | 12.5±8.6 | 9.9±7.8 | 0.2 |
| AF/AFL history | 13.0±9.0 | 9.9±8.0 | 0.2 | 12.9±8.9 | 10.0±8.0 | 0.2 | 12.5±8.6 | 9.9±7.8 | 0.2 |
| TIA/stroke history | 13.0±9.0 | 9.9±8.0 | -- | 12.9±8.9 | 10.0±8.0 | -- | 12.5±8.6 | 9.9±7.8 | -- |

Abbrev. HbA1c, hemoglobulin A1c; TG, triglyceride; TCHO, total cholesterol; LDL, low density lipoprotein; HDL, high density lipoprotein; AST, aspartate aminotransferase; ALT, alanine aminotransferase; eGFR, estimated glomerular filtration rate; hsCRP, high-sensitivity C-reactive protein; Hct, hematocrit; NT-proBNP, N-terminal pro-B-type natriuretic peptide; BNP, brain natriuretic peptide; BMI, body mass index; LVIDd, left ventricular internal diameter end diastole; LVIDs, left ventricular internal diameter end systole; LV mass, left ventricular mass; LA, left atrial size; E, maximal early diastolic transmitral flow velocity; A, maximal late diastolic transmitral flow velocity; DT, mitral flow deceleration time; TR max PG, maximal pressure gradient of tricuspid regurgitation; E’, maximal early diastolic mitral annular velocity; A’, maximal late diastolic mitral annular velocity; LVEF, left ventricular ejection fraction; COPD, chronic obstructive pulmonary disease; AMI, acute myocardial infarction; PAOD, peripheral arterial occlusive disease; DM, diabetes mellitus; HF, heart failure; AF/AFL, atrial fibrillation/atrial flutter; TIA, transient ischemic attack.

**Supplemental Table 2.** Hyperparameters

|  | Hyperparameters |
| --- | --- |
| LR | We used the default settings suggested by scikit-learn [s1]. |
| SVM |  |
| RF | We tuned hyperparameters around the models’ default settings to maximize the average primary metric (AUPRC) in 3-fold cross-validation on the training set.  The optimal hyperparameters vary in different experimental settings (imputation types, time-series or not, etc.) |
| XGB |  |
| Hyperparameters shared by all deep learning models | Optimizer: Adam  Learning rate: 1e^-3^  Batch size: 32  Regularization: L2-norm (coefficient = 1e^-4^)  Earlystopping patience: 40 |
| GRU | Dimension of the GRU layer: 30  Dimension of the fully-connected layer: 20 |
| ForeSIIN  (with a shared fully-connected layer) | Dimension of each independent GRU layer: 5  Dimension of the shared fully-connected layer: 15 |
| **ForeSIIN**  **(with independent fully-connected layers)** | Dimension of each independent GRU output: 5  Dimension of each independent fully-connected layer: 10 |

[s1] Pedregosa, F., Varoquaux, G., Gramfort, A., Michel, V., Thirion, B., Grisel, O., ... & Duchesnay, É. (2011). Scikit-learn: Machine learning in Python. *the Journal of machine Learning research*, *12*, 2825-2830.

**Supplemental Table 3.** Detailed performance comparison on the independent test set.

|  | Input: single time step | | Input: time series  (Flattened for non-sequence models) | |
| --- | --- | --- | --- | --- |
|  | AUROC | AUPRC  (baseline: 0.038) | AUROC | AUPRC  (baseline: 0.038) |
| CHA_2_DS_2_-VASc score | 0.650 (0.596, 0.699) | 0.103 (0.040, 0.142) | -- | -- |
| ATRIA | 0.703 (0.657, 0.754) | 0.143 (0.073, 0.201) | -- | -- |
| LR  (mean/mode imputation) | 0.508 (0.459, 0.561) | 0.037 (0.027, 0.045) | 0.580 (0.529, 0.633) | 0.047 (0.035, 0.059) |
| LR  (no imputation) | 0.514 (0.468, 0.561) | 0.038 (0.029, 0.046) | 0.544 (0.498, 0.591) | 0.041 (0.031, 0.50) |
| SVM  (mean/mode imputation) | 0.650 (0.592, 0.706) | 0.134 (0.074, 0.186) | 0.606 (0.548, 0.667) | 0.137 (0.067, 0.195) |
| SVM  (no imputation) | 0.691 (0.637, 0.741) | 0.153 (0.083, 0.210) | 0.670 (0.577, 0.681) | 0.145 (0.078, 0.206) |
| RF  (mean/mode imputation) | 0.689 (0.637, 0.742) | 0.169 (0.095, 0.230) | 0.641 (0.585, 0.701) | 0.139 (0.077, 0.194) |
| RF  (no imputation) | 0.703 (0.648, 0.756) | 0.135 (0.074, 0.186) | 0.632 (0.570, 0.692) | 0.124 (0.060, 0.174) |
| XGB  (mean/mode imputation) | 0.714 (0.668, 0.759) | 0.133 (0.079, 0.179) | 0.703 (0.657, 0.749) | 0.131 (0.067, 0.180) |
| XGB  (no imputation) | 0.722 (0.676, 0.769) | 0.144 (0.077, 0.196) | 0.694 (0.645, 0.741) | 0.099 (0.056, 0.132) |
| GRU  (mean/mode imputation) | -- | -- | 0.511 (0.485, 0.533) | 0.035 (0.022, 0.046) |
| GRU  (no imputation) | -- | -- | 0.633 (0.581, 0.691) | 0.071 (0.045, 0.091) |
| **ForeSIIN**  **(mean/mode imputation)** | -- | -- | 0.696 (0.647, 0.744) | 0.149 (0.087, 0.204) |
| **ForeSIIN**  **(no imputation)** | -- | -- | 0.764 (0.722, 0.810) | 0.210 (0.137, 0.283) |

|  | Input: single time step | | Input: time series  (Flattened for non-sequence models) | |
| --- | --- | --- | --- | --- |
|  | F1-score | Youden statistic | F1-score | Youden statistic |
| CHA_2_DS_2_-VASc score | 0.112 (0.041, 0.181) | 0.223 (0.132, 0.302) | -- | -- |
| ATRIA | 0.134 (0.056, 0.204) | 0.262 (0.166, 0.356) | -- | -- |
| LR  (mean/mode imputation) | 0.075 (0.055, 0.094) | 0.032 (-0.046, 0.125) | 0.000 (0.000, 0.000) | 0.117 (0.035, 0.205) |
| LR  (no imputation) | 0.080 (0.064, 0.093) | 0.075 (0.021, 0.134) | 0.066 (0.036, 0.093) | 0.04 (-0.057, 0.077) |
| SVM  (mean/mode imputation) | 0.278 (0.199, 0.360) | 0.244 (0.161, 0.327) | 0.248 (0.168, 0.329) | 0.240 (0.161, 0.320) |
| SVM  (no imputation) | 0.251 (0.175, 0.328) | 0.260 (0.171, 0.351) | 0.201 (0.145, 0.257) | 0.209 (0.124, 0.287) |
| RF  (mean/mode imputation) | 0.262 (0.188, 0.336) | 0.253 (0.171, 0.337) | 0.243 (0.169, 0.316) | 0.229 (0.141, 0.325) |
| RF  (no imputation) | 0.167 (0.100, 0.227) | 0.331 (0.236, 0.421) | 0.175 (0.097, 0.247) | 0.220 (0.133, 0.310) |
| XGB  (mean/mode imputation) | 0.201 (0.127, 0.279) | 0.237 (0.161, 0.317) | 0.226 (0.151, 0.302) | 0.186 (0.110, 0.262) |
| XGB  (no imputation) | 0.190 (0.111, 0.260) | 0.311 (0.221, 0.399) | 0.149 (0.084, 0.215) | 0.208 (0.126, 0.286) |
| GRU  (mean/mode imputation) | -- | -- | 0.036 (0.005, 0.060) | -0.019 (-0.063, 0.019) |
| GRU  (no imputation) | -- | -- | 0.144 (0.102, 0.183) | 0.160 (0.067, 0.253) |
| **ForeSIIN**  **(mean/mode imputation)** | -- | -- | 0.254 (0.184, 0.328) | 0.304 (0.216, 0.393) |
| **ForeSIIN**  **(no imputation)** | -- | -- | 0.280 (0.209, 0.356) | 0.373 (0.279, 0.463) |

**Supplemental Table 4.** ForeSIIN with four types of input feature set: (1) 39 features other than the dominant feature (stroke history) (2) the dominant feature only (3) top-10 impactful features identified from the feature impact difference between correctly predicted high/low-risk patients (4) all features

|  | Input: time series  (Flattened for non-sequence models) | |
| --- | --- | --- |
|  | AUROC | AUPRC  (baseline: 0.038) |
| ForeSIIN  (with 39 features other than the stroke history) | 0.628 (0.582, 0.676) | 0.056 (0.040, 0.069) |
| ForeSIIN  (with the stroke history only) | 0.622 (0.560, 0.682) | 0.134 (0.081, 0.180) |
| ForeSIIN  (with top-10 impactful features) | 0.726 (0.678, 0.774) | 0.162 (0.083, 0.220) |
| **ForeSIIN**  **(all features)** | 0.764 (0.722, 0.810) | 0.210 (0.137, 0.283) |
|  | Input: time series  (Flattened for non-sequence models) | |
|  | F1-score | Youden index |
| ForeSIIN  (with 39 features other than the stroke history) | 0.103 (0.080, 0.124) | 0.199 (0.108, 0.292) |
| ForeSIIN  (with the stroke history only) | 0.288 (0.215, 0.360) | 0.266 (0.186, 0.343) |
| ForeSIIN  (with top-10 impactful features) | 0.241 (0.151, 0.325) | 0.295 (0.201, 0.389) |
| **ForeSIIN**  **(all features)** | 0.280 (0.209, 0.356) | 0.373 (0.279, 0.463) |

**Supplemental Table 5.** Performance comparison between two ForeSIIN structures

|  | Input: time series  (Flattened for non-sequence models) | |
| --- | --- | --- |
|  | AUROC | AUPRC  (baseline: 0.038) |
| ForeSIIN  (with a shared fully-connected layer) | 0.756 (0.715, 0.799) | 0.174 (0.100, 0.231) |
| **ForeSIIN**  **(with independent fully-connected layers)** | 0.764 (0.722, 0.810) | 0.210 (0.137, 0.283) |
|  | Input: time series  (Flattened for non-sequence models) | |
|  | F1-score | Youden index |
| ForeSIIN  (with a shared fully-connected layer) | 0.265 (0.176, 0.342) | 0.368 (0.285, 0.456) |
| **ForeSIIN**  **(with independent fully-connected layers)** | 0.280 (0.209, 0.356) | 0.373 (0.279, 0.463) |

**Supplemental Table 6.** Detailed 3-fold cross-validation performance comparison of National Taiwan University Hospital. The number in the parentheses denotes the standard deviation.

|  | AUPRC  (std) | AUROC  (std) | Youden index  (std) | F1-score  (std) |
| --- | --- | --- | --- | --- |
| CHA_2_DS_2_-VASc score | 0.099 (0.012) | 0.652 (0.006) | 0.141 (0.051) | 0.074 (0.015) |
| ATRIA score | 0.133 (0.017) | 0.689 (0.026) | 0.138 (0.029) | 0.126 (0.054) |
| LR^*,++^ | 0.042 (0.005) | 0.536 (0.041) | 0.065 (0.049) | 0.077 (0.007) |
| SVM^**, +^ | 0.131 (0.010) | 0.678 (0.014) | 0.248 (0.030) | 0.227 (0.026) |
| RF^*,+^ | 0.147 (0.037) | 0.680 (0.036) | 0.272 (0.055) | **0.237 (0.058)** |
| XGB^**, +^ | 0.127 (0.025) | 0.710 (0.022) | 0.276 (0.041) | 0.192 (0.053) |
| GRU^**, ++^ | 0.067 (0.008) | 0.627 (0.006) | 0.145 (0.023) | 0.100 (0.015) |
| **ForeSIIN**^*, ++^ | – | – | – | – |
| **ForeSIIN**^**, ++^ | **0.156 (0.041)** | **0.738 (0.041)** | **0.294 (0.042)** | 0.234 (0.026) |

*: mean/mode imputation.

**: no imputation (replace with -1).

+: only input the last summarization interval containing the first AF event.

++: input the complete input sequence (flattened in the non-sequential model case).

Abbrev. LR, logistic regression; SVM, support vector machine; RF, random forest; XGB, XGBoost; GRU, gated recurrent units.

**Supplemental Table 7.** Detailed performance comparison on the external validation dataset. The external validation dataset was collected from the National Taiwan University Hospital-Yunlin Branch with the same criteria, having a sample size of 6682 AF patients.

|  | AUPRC  (95% CI) | AUROC  (95%CI) | Youden index  (95%CI) | F1-score  (95%CI) |
| --- | --- | --- | --- | --- |
| CHA_2_DS_2_-VASc score | 0.132 (0.106, 0.155) | 0.599 (0.572, 0.625) | 0.133 (0.100, 0.164) | 0.181 (0.155, 0.207) |
| ATRIA score | 0.132 (0.107, 0.153) | 0.610 (0.584, 0.635) | 0.146 (0.104, 0.192) | 0.169 (0.150, 0.185) |
| LR^*,++^ | 0.078 (0.068, 0.087) | 0.501 (0.475, 0.527) | 0.003 (0.000, 0.018) | 0.045 (0.023, 0.065) |
| SVM^**, +^ | 0.199 (0.165, 0.230) | **0.650 (0.625, 0.678)** | 0.191 (0.145, 0.234) | 0.238 (0.206, 0.270) |
| RF^*,+^ | 0.179 (0.146, 0.208) | 0.626 (0.598, 0.654) | **0.221 (0.177, 0.263)** | 0.247 (0.211, 0.284) |
| XGB^**, +^ | 0.185 (0.154, 0.216) | 0.643 (0.615, 0.670) | 0.181 (0.137, 0.225) | 0.255 (0.218, 0.294) |
| GRU^**, ++^ | 0.093 (0.081, 0.103) | 0.581 (0.557, 0.603) | 0.148 (0.105, 0.191) | 0.077 (0.052, 0.100) |
| **ForeSIIN**^*, ++^ | 0.210 (0.171, 0.247) | 0.605 (0.576, 0.634) | 0.164 (0.148, 0.178) | 0.264 (0.220, 0.308) |
| **ForeSIIN**^**, ++^ | **0.222 (0.184, 0.259)** | 0.646 (0.618, 0.671) | 0.179 (0.164, 0.192) | **0.282 (0.239, 0.324)** |

*: mean/mode imputation.

**: no imputation (replace with -1).

+: only input the last summarization interval containing the first AF event.

++: input the complete input sequence (flattened in the non-sequential model case).

Abbrev. LR, logistic regression; SVM, support vector machine; RF, random forest; XGB, XGBoost; GRU, gated recurrent units.

**Supplemental Table 8.** Detailed mean feature impacts of patient groups t0, t1, and their difference.

|  | Label 0  (ground truth) | Label 1  (ground truth) | Difference  (Label 1 - Label 0) |
| --- | --- | --- | --- |
| TIA/stroke history | -1.7796 | -0.6927 | 1.086877 |
| eGFR | 0.01487 | 0.19562 | 0.18075 |
| hsCRP | -0.0573 | 0.02587 | 0.083182 |
| Hct | 0.28507 | 0.36381 | 0.078747 |
| Glucose | -0.1614 | -0.1157 | 0.045673 |
| E’ | 0.08832 | 0.12145 | 0.033126 |
| Hypertension history | -0.0063 | -0.0329 | -0.02667 |
| LVEF | -0.0621 | -0.0362 | 0.02586 |
| LV mass | 0.10602 | 0.12877 | 0.022747 |
| ALT | -0.0402 | -0.0193 | 0.020805 |
| BMI | -0.0996 | -0.1193 | -0.01978 |
| A | -0.0783 | -0.0933 | -0.01496 |
| LDL | -0.0332 | -0.0476 | -0.01439 |
| Age | -0.0905 | -0.0801 | 0.010398 |
| HbA1C | -0.0028 | -0.012 | -0.00916 |
| AF/AFL | -0.0358 | -0.0439 | -0.00806 |
| TG | -0.0707 | -0.064 | 0.006686 |
| TR max PG | -0.0073 | -0.003 | 0.004352 |
| AST | 0.00912 | 0.01342 | 0.004299 |
| Hyperlipidemia history | -0.0177 | -0.0211 | -0.00343 |
| Gender | -0.0481 | -0.0448 | 0.003246 |
| E | -0.0265 | -0.0236 | 0.002897 |
| TCHO | -0.0011 | -0.0033 | -0.00219 |
| BNP | -0.1434 | -0.145 | -0.00168 |
| Mitral regurgitation | -0.0263 | -0.0275 | -0.00128 |
| NT-proBNP | -0.0117 | -0.0125 | -0.0008 |
| PAOD history | -0.0117 | -0.0125 | -0.00077 |
| LVIDd | -0.0161 | -0.0167 | -0.00054 |
| LVIDs | -0.0134 | -0.0139 | -0.00049 |
| E/A ratio | -0.0282 | -0.0286 | -0.00038 |
| HDL | -0.0123 | -0.0125 | -0.00026 |
| DM history | -0.0117 | -0.0114 | 0.00024 |
| LA size | -0.0134 | -0.0136 | -0.00014 |
| HF history | -0.0093 | -0.0094 | -0.00014 |
| COPD history | -0.0138 | -0.0137 | 0.000102 |
| A’ | -0.004 | -0.0039 | 4.09E-05 |
| Aortic regurgitation | -0.0091 | -0.0092 | -3.80E-05 |
| AMI history | -0.0144 | -0.0144 | -3.34E-05 |
| DT | -0.0111 | -0.0111 | -2.87E-05 |
| Gout history | -0.0146 | -0.0146 | -1.08E-05 |

Abbrev. TIA, transient ischemic attack; eGFR, estimated glomerular filtration rate; hsCRP, high-sensitivity C-reactive protein; Hct, hematocrit; E’, maximal early diastolic mitral annular velocity; HTN, hypertension; LVEF, left ventricular ejection fraction; LV mass, left ventricular mass; ALT, alanine aminotransferase; BMI, body mass index; A, maximal late diastolic transmitral flow velocity; LDL, low-density lipoprotein; HbA1c, hemoglobulin A1c; AF/AFL, atrial fibrillation/atrial flutter; TG, triglyceride; TR max PG, maximal pressure gradient of tricuspid regurgitation; AST, aspartate aminotransferase; E, maximal early diastolic transmitral flow velocity; TCHO, total cholesterol; BNP, brain natriuretic peptide; NT-proBNP, N-terminal pro-B-type natriuretic peptide; PAOD, peripheral arterial occlusive disease; LVIDd, left ventricular internal diameter end diastole; LVIDs, left ventricular internal diameter end systole; HDL, high density lipoprotein; DM, diabetes mellitus; LA, left atrial size; HF, heart failure, COPD, chronic obstructive pulmonary disease; A’, maximal late diastolic mitral annular velocity; AMI, acute myocardial infarction; DT, mitral flow deceleration time.

**Supplemental Table 9.** Detailed mean feature impacts of correctly classified patient groups t0p0, t1p1, and their difference.

|  | Label 0  (correctly predicted) | Label 1  (correctly predicted) | Difference  (Label 1 - Label 0) |
| --- | --- | --- | --- |
| TIA/stroke history | -1.8613 | 0.02704 | 1.88833 |
| eGFR | -0.0734 | 0.1901 | 0.263449 |
| hsCRP | -0.0943 | 0.02038 | 0.114679 |
| Hct | 0.23637 | 0.33433 | 0.097957 |
| Glucose | -0.1904 | -0.1152 | 0.075179 |
| E’ | 0.06685 | 0.13743 | 0.070585 |
| LVEF | -0.083 | -0.0334 | 0.049621 |
| LV mass | 0.07785 | 0.12609 | 0.048239 |
| ALT | -0.0523 | -0.0232 | 0.029095 |
| HbA1C | -0.0191 | 0.00817 | 0.027221 |
| Gender | -0.0609 | -0.0354 | 0.025496 |
| Age | -0.0995 | -0.0751 | 0.024464 |
| LDL | -0.0285 | -0.0514 | -0.02291 |
| BMI | -0.104 | -0.1184 | -0.01442 |
| A | -0.0768 | -0.09 | -0.01319 |
| BNP | -0.1472 | -0.1352 | 0.01197 |
| TR max PG | -0.0112 | -0.0021 | 0.009094 |
| TG | -0.0717 | -0.0632 | 0.008432 |
| AST | 0.00711 | 0.01249 | 0.005374 |
| Hyperlipidemia | -0.02 | -0.0236 | -0.00357 |
| E | -0.0277 | -0.0242 | 0.003493 |
| Mitral regurgitation | -0.0257 | -0.0276 | -0.00196 |
| Hypertension | -0.0351 | -0.037 | -0.00193 |
| AF/AFL history | -0.0364 | -0.0354 | 0.001008 |
| LVIDd | -0.0158 | -0.0168 | -0.00099 |
| LVIDs | -0.0132 | -0.0141 | -0.0009 |
| NT-proBNP | -0.0113 | -0.012 | -0.00076 |
| TCHO | 0.00245 | 0.00178 | -0.00067 |
| PAOD history | -0.012 | -0.0126 | -0.00064 |
| HDL | -0.0121 | -0.0126 | -0.00051 |
| LA size | -0.0134 | -0.0136 | -0.00024 |
| E/A ratio | -0.028 | -0.0282 | -0.00021 |
| Aortic regurgitation | -0.0091 | -0.0093 | -0.0002 |
| HF history | -0.0093 | -0.0095 | -0.00018 |
| COPD history | -0.0138 | -0.0136 | 0.000155 |
| A’ | -0.004 | -0.0039 | 5.23E-05 |
| DT | -0.0111 | -0.0111 | -4.16E-05 |
| AMI history | -0.0144 | -0.0144 | -4.12E-05 |
| DM history | -0.0112 | -0.0112 | 3.71E-05 |
| Gout history | -0.0146 | -0.0146 | -1.57E-05 |

Abbrev. TIA, transient ischemic attack; eGFR, estimated glomerular filtration rate; hsCRP, high sensitivity C reactive protein; Hct, hematocrit; E’, maximal early diastolic mitral annular velocity; LVEF, left ventricular ejection fraction; LV mass, left ventricular mass; ALT, alanine aminotransferase; HbA1c, hemoglobulin A1c; LDL, low density lipoprotein; BMI, body mass index; A, maximal late diastolic transmitral flow velocity; BNP, brain natriuretic peptide; TR max PG, maximal pressure gradient of tricuspid regurgitation; TG, triglyceride; AST, aspartate aminotransferase; E, maximal early diastolic transmitral flow velocity; AF/AFL, atrial fibrillation/atrial flutter; LVIDd, left ventricular internal diameter end diastole; LVIDs, left ventricular internal diameter end systole; NT-proBNP, N-terminal pro-B-type natriuretic peptide; TCHO, total cholesterol; PAOD, peripheral arterial occlusive disease; HDL, high density lipoprotein; LA, left atrial size; HF, heart failure, COPD, chronic obstructive pulmonary disease; A’, maximal late diastolic mitral annular velocity; DT, mitral flow deceleration time; AMI, acute myocardial infarction; DM, diabetes mellitus.

**Supplemental Table 10.** Detailed mean feature impacts of misclassified patient groups t0p1and t1p0

|  | Label 0  (missed) | Label 1  (missed) |
| --- | --- | --- |
| Age | -0.06409 | -0.0886 |
| HbA1C | 0.04478 | -0.04581 |
| Glucose | -0.07632 | -0.11647 |
| TG | -0.06796 | -0.06539 |
| TCHO | -0.01136 | -0.01174 |
| LDL | -0.04722 | -0.04134 |
| HDL | -0.01273 | -0.01238 |
| AST | 0.014984 | 0.014978 |
| ALT | -0.0045 | -0.01281 |
| eGFR | 0.273209 | 0.2049 |
| hsCRP | 0.050994 | 0.035097 |
| Hct | 0.427671 | 0.413402 |
| NT-proBNP | -0.01278 | -0.01317 |
| BNP | -0.1321 | -0.16152 |
| BMI | -0.08647 | -0.12083 |
| LVIDd | -0.01702 | -0.01642 |
| LVIDs | -0.01422 | -0.01369 |
| LV mass | 0.188516 | 0.133271 |
| LA size | -0.01364 | -0.01353 |
| E | -0.02302 | -0.0226 |
| A | -0.08274 | -0.0988 |
| E/A ratio | -0.02867 | -0.02912 |
| DT | -0.01111 | -0.0111 |
| TR max PG | 0.003904 | -0.0045 |
| E’ | 0.15121 | 0.094565 |
| A’ | -0.00392 | -0.00391 |
| LVEF | -0.00063 | -0.04089 |
| Hypertension history | 0.078061 | -0.02611 |
| Hyperlipidemia history | -0.01086 | -0.01698 |
| Gout history | -0.01454 | -0.01455 |
| COPD history | -0.01399 | -0.01392 |
| AMI history | -0.01432 | -0.01436 |
| PAOD history | -0.01087 | -0.01222 |
| Mitral regurgitation | -0.0279 | -0.02733 |
| Aortic regurgitation | -0.00937 | -0.00905 |
| Gender | -0.0104 | -0.06062 |
| DM history | -0.0129 | -0.01179 |
| HF history | -0.0091 | -0.00925 |
| AF/AFL history | -0.03422 | -0.05822 |
| TIA/stroke history | -1.54029 | -1.90317 |

Abbrev. HbA1c, hemoglobulin A1c; TG, triglyceride; TCHO, total cholesterol; LDL, low-density lipoprotein; HDL, high-density lipoprotein; AST, alanine aspartate aminotransferase; ALT, alanine aminotransferase; eGFR, estimated glomerular filtration rate; hsCRP, high sensitive C-reactive protein; Hct, hematocrit; NT-proBNP, N-terminal pro-B-type natriuretic peptide; BNP, brain natriuretic peptide; BMI, body mass index; LVIDd, left ventricular internal diameter end diastole; LVIDs, left ventricular internal diameter end systole; LV mass, left ventricular mass; LA, left atrial size; E, maximal early diastolic transmitral flow velocity; A, maximal late diastolic transmitral flow velocity; DT, mitral flow deceleration time; TR max PG, maximal pressure gradient of tricuspid regurgitation. E’, maximal early diastolic mitral annular velocity; A’, maximal late diastolic mitral annular velocity; LVEF, left ventricular ejection fraction; COPD, chronic obstructive pulmonary disease; AMI, acute myocardial infarction; PAOD, peripheral arterial occlusive disease; DM, diabetes mellitus; HF, heart failure; AF/AFL, atrial fibrillation/atrial flutter; TIA, transient ischemic attack.

**Supplemental Table 11.** Detailed mean feature impacts of correctly predicted patient groups t0p0h1 and t1p1h0, which are relatively difficult to classify

|  | Label 0  (correctly predicted with a stroke history) | Label 1  (correctly predicted without a stroke history) |
| --- | --- | --- |
| Age | -0.08833 | -0.06582 |
| HbA1C | -0.18706 | 0.031176 |
| Glucose | -0.30793 | -0.03282 |
| TG | -0.04869 | -0.06034 |
| TCHO | -0.02263 | -0.00643 |
| LDL | -0.02477 | -0.0588 |
| HDL | -0.01096 | -0.01291 |
| AST | 0.000427 | 0.027069 |
| ALT | -0.09762 | -0.00211 |
| eGFR | -0.62561 | 0.346236 |
| hsCRP | -0.43414 | 0.05904 |
| Hct | -0.37775 | 0.468658 |
| NT-proBNP | -0.00848 | -0.01308 |
| BNP | -0.16152 | -0.14454 |
| BMI | -0.12358 | -0.10383 |
| LVIDd | -0.01547 | -0.01708 |
| LVIDs | -0.01323 | -0.01428 |
| LV mass | -0.54015 | 0.201033 |
| LA size | -0.01324 | -0.01368 |
| E | -0.03375 | -0.02277 |
| A | -0.1129 | -0.0755 |
| E/A ratio | -0.02837 | -0.02824 |
| DT | -0.01102 | -0.01112 |
| TR max PG | -0.04395 | 0.003988 |
| E’ | 0.010084 | 0.136476 |
| A’ | -0.00397 | -0.00391 |
| LVEF | -0.37393 | 0.003944 |
| Hypertension history | -0.10995 | 0.107347 |
| Hyperlipidemia history | -0.03634 | -0.0117 |
| Gout history | -0.01462 | -0.01454 |
| COPD history | -0.0133 | -0.01405 |
| AMI history | -0.01452 | -0.01433 |
| PAOD history | -0.01442 | -0.01082 |
| Mitral regurgitation | -0.02549 | -0.0281 |
| Aortic regurgitation | -0.00906 | -0.00937 |
| Gender | -0.08178 | -0.01458 |
| DM history | -0.00884 | -0.013 |
| HF history | -0.00998 | -0.00906 |
| AF/AFL history | -0.04112 | -0.01851 |
| TIA/stroke history | 1.644202 | -1.96239 |

Abbrev. HbA1c, hemoglobulin A1c; TG, triglyceride; TCHO, total cholesterol; LDL, low-density lipoprotein; HDL, high-density lipoprotein; AST, alanine aspartate aminotransferase; ALT, alanine aminotransferase; eGFR, estimated glomerular filtration rate; hsCRP, high sensitive C-reactive protein; Hct, hematocrit; NT-proBNP, N-terminal pro-B-type natriuretic peptide; BNP, brain natriuretic peptide; BMI, body mass index; LVIDd, left ventricular internal diameter end diastole; LVIDs, left ventricular internal diameter end systole; LV mass, left ventricular mass; LA, left atrial size; E, maximal early diastolic transmitral flow velocity; A, maximal late diastolic transmitral flow velocity; DT, mitral flow deceleration time; TR max PG, maximal pressure gradient of tricuspid regurgitation. E’, maximal early diastolic mitral annular velocity; A’, maximal late diastolic mitral annular velocity; LVEF, left ventricular ejection fraction; COPD, chronic obstructive pulmonary disease; AMI, acute myocardial infarction; PAOD, peripheral arterial occlusive disease; DM, diabetes mellitus; HF, heart failure; AF/AFL, atrial fibrillation/atrial flutter; TIA, transient ischemic attack.
